# Phase I/II study of docetaxel and S-1 in patients with advanced gastric cancer

**DOI:** 10.1038/sj.bjc.6603196

**Published:** 2006-06-13

**Authors:** K Yamaguchi, T Shimamura, I Hyodo, W Koizumi, T Doi, H Narahara, Y Komatsu, T Kato, S Saitoh, T Akiya, M Munakata, Y Miyata, Y Maeda, H Takiuchi, S Nakano, T Esaki, F Kinjo, Y Sakata

**Affiliations:** 1 Department of Gastroenterology, Saitama Cancer Centre, 818 Komuro, Ina-machi, Kitaadachi-gun, Saitama 362-0806, Japan; 2 Division of Gastroenterology, Institute of Medicine, University of Tsukuba, Ibaraki, Japan; 3 Department of Gastroenterology and Medical Oncology, Kitasato University East Hospital, Kanagawa, Japan; 4 Division of Digestive Endoscopy and Gastrointestinal Oncology, National Cancer Centre Hospital East, Chiba, Japan; 5 Department of Clinical Oncology, Hiroshima University Graduate School of Medicine, Hiroshima, Japan; 6 Department of Medical Oncology, Hokkaido University Graduate School of Medicine, Hokkaido, Japan; 7 Division of Internal Medicine, Niigata Cancer Centre Hospital, Niigata, Japan; 8 Department of Gastroenterology, Aomori Prefectural Centre Hospital, Aomori, Japan; 9 Department of Internal Medicine, Gunma Prefectural Cancer Centre, Gunma, Japan; 10 Department of Internal Medicine, Misawa Municipal Hospital, Aomori, Japan; 11 Department of Gastroenterology, Saku Central Hospital, Nagano, Japan; 12 Department of Chemotherapy, Tokyo Metropolitan Komagome Hospital, Tokyo, Japan; 13 Second Department of Internal Medicine, Osaka Medical College, Osaka, Japan; 14 First Department of Internal Medicine and Department of Medicine and Biosystemic Science, Graduate School of Medicine, Kyushu University, Fukuoka, Japan; 15 Department of Cancer Chemotherapy, National Kyushu Cancer Centre, Fukuoka, Japan; 16 Department of Endoscopy, Faculty of Medicine, University of the Ryukyu, Okinawa, Japan

**Keywords:** advanced gastric cancer, docetaxel, S-1, clinical trial, phase I/II

## Abstract

The aims of this phase I/II study of docetaxel and S-1 were to determine the dose-limiting toxicity (DLT), maximum-tolerated dose (MTD), and recommended dose (RD) in the phase I part and to explore the tumour response, survival and safety in the phase II part. Patients with histologically- or cytologically confirmed unresectable or recurrent gastric cancer were eligible. Treatment consisted of intravenous docetaxel on day 1 (starting dose 50 mg m^−2^) and oral S-1 at a fixed dose of 40 mg m^−2^ twice daily on days 1–14, every 4 weeks up to six cycles. Nine patients took part in the phase I portion of the study. The MTD of docetaxel was determined to be 50 mg m^−2^, with the DLTs of grade 3 infection associated with grade 3 neutropenia and grade 4 neutropenia during S-1 administration. The RD of docetaxel was 40 mg m^−2^ in combination with S-1 40 mg m^−2^ b.i.d. The efficacy and safety of this regimen was therefore assessed in 46 patients with at least one measurable lesion. The overall response rate and estimated median overall survival were 46% (95% CI, 31–61%) and 14.0 months (8.3–17.3 months), respectively. The most common grade 3/4 toxicity was neutropenia (67% of patients), which was predictable and manageable. This regimen showed promising activity with moderate toxicities in advanced gastric cancer.

Although the incidence of gastric cancer (GC) has been declining, it remains one of the most common causes of cancer-related deaths (Jemal *et al*, 2005). Surgical resection at the early stage of disease is considered the most important intervention that leads to long-term disease-free survival, but many patients have recurrences or are diagnosed with more advanced stages of disease. For such patients, 1-year survival rates are approximately 50% in stage III disease, and <25% in stage IV disease (Alberts *et al*, 2003). Thus, considerable attention has been paid to the development of effective treatment for patients with advanced GC.

Cytotoxic chemotherapy including 5-fluorouracil (5-FU) is the most effective means of providing survival benefits and improvements in quality of life compared with best supportive care (Wöhrer *et al*, 2004). However, these improvements have been modest, at best, and thus far no combination has clearly provided a survival advantage over single agent 5-FU (Ohtsu, 2005). Therefore, although no accepted global standard regimen has been established, most physicians rely on 5-FU as monotherapy or as part of a combination strategy.

Docetaxel (Taxotere®, sanofi-aventis, Paris, France) is a semisynthetic taxoid derived from the European yew tree, *Taxus baccata* (Denis *et al*, 1990). A preclinical study of docetaxel showed a synergistic antitumour activity in combination with 5-FU (Bissery *et al*, 1995), which led to several clinical studies in advanced GC. In this setting, docetaxel has been evaluated both as a single agent (Sulkes *et al*, 1994; Einzig *et al*, 1996; Taguchi *et al*, 1998; Mai *et al*, 1999) and in combination with fluoropyrimidines (Constenla *et al*, 2002; Park *et al*, 2004), cisplatin (Roth *et al*, 2000), and cisplatin plus 5-FU (Van Custem *et al*, 2003). In a randomised phase III study, this latter regimen (TCF) improved overall survival compared to cisplatin plus 5-FU (Moiseyenko *et al*, 2005). The docetaxel containing combination regimens are associated with severe leucopenia and febrile neutropenia, and although these adverse events can be managed, efforts must be made to minimise such toxicities (Sulkes *et al*, 1994; Einzig *et al*, 1996; Taguchi *et al*, 1998; Mai *et al*, 1999).

The anticancer drug S-1 (TS-1®, Taiho Pharmaceutical Co. Ltd., Tokyo, Japan) is an oral formulation containing within each capsule tegafur, a 5-FU prodrug; gimeracil, an inhibitor of dihydropyrimidine dehydrogenase; and oteracil, which inhibits pyrimidine phosphoribosyl transferase specifically in the gastrointestinal tract and thereby decreases the phosphorylation of 5-FU in the intestine (Shirasaka *et al*, 1996). Phase II studies of S-1 monotherapy in patients with advanced GC showed overall response rates (ORRs) of 26–49% with the most relevant side-effects being diarrhoea in a European study and neutropenia in two Japanese studies (Sakata *et al*, 1998; Koizumi *et al*, 2000; Chollet *et al*, 2003).

Based on the clinical activity of both docetaxel and S-1, as well as the promising efficacy of docetaxel when combined with other fluoropyrimidines, we conducted a phase I/II study of docetaxel and S-1 in order to develop an effective treatment for patients with advanced GC that would improve on the safety profile of earlier taxane-fluoropyrimidine combinations.

## MATERIALS AND METHODS

### Study design

This was a multicentre, open-label, single-arm, phase I/II study conducted at 16 institutions in Japan. The objective of the phase I part was to determine the dose-limiting toxicity (DLT), maximum-tolerated dose (MTD) and recommended dose (RD) of docetaxel combined with a fixed dose of S-1. In the phase II part, the primary objective was to estimate the ORR of this combination at the RD. Secondary objectives were to assess progression-free survival, overall survival, 1-year survival rate, and adverse events. This study was conducted in accordance with the principles of the Declaration of Helsinki and Good Clinical Practice guidelines. The study protocol was reviewed and approved by the institutional review board of each participating centre. The objective response was independently reviewed by the extramural review committee, and the MTD and RD were determined by the independent data monitoring committee.

### Patients

Patients were eligible if they signed informed consent and met all the following criteria: histologically- or cytologically confirmed unresectable or recurrent GC; at least one measurable lesion (patients without a measurable lesion, but evaluable nonmeasurable lesions were permitted in the phase I part of this study); Eastern Cooperative Oncology Group performance status of 0–1; good recovery from surgery (at least 28 days after the operation); up to one prior chemotherapy regimen except for prior taxane (paclitaxel or docetaxel) or S-1 (other fluoropyrimidines were allowed if at least 28 days elapsed after the last treatment); ⩾20 years of age; estimated life expectancy of at least 3 months; haemoglobin ⩾8.0 g dl^−1^; white blood cell count between 4000 and 12 000 mm^−3^; neutrophil count ⩾2000 mm^−3^; platelet count ⩾100 000 mm^−3^; serum bilirubin ⩽1.5 mg dl^−1^; aspartate aminotransferase and alanine aminotransferase ⩽2.5 times the upper limit of normal (ULN); and serum creatinine less than or equal to ULN.

Exclusion criteria were as follows: pregnant female or sexually active males/females unwilling to use contraception during the study; infection or suspected infection with fever; congestive heart failure; uncontrolled angina pectoris or arrhythmia; a history of myocardial infarction within the previous 3 months; uncontrolled diabetes or hypertension; interstitial pneumonia or lung fibrosis; peripheral neuropathy grade 2 or higher; pleural, peritoneal, or pericardial effusion that required treatment; gastrointestinal haemorrhage; symptomatic brain metastasis; diarrhoea; and active concomitant malignancy.

### Phase I part

Patients received variable doses of intravenous docetaxel administered as a 1–2-h infusion on day 1 and oral S-1 administered at a fixed dose of 40 mg m^−2^ twice daily on days 1–14 every 4 weeks (one cycle). Patients were treated for up to six cycles unless disease progression or unacceptable toxicity was observed.

The initial starting dose of docetaxel was 50 mg m^−2^ (dose level 1), and step-wise dose increases to 60 and 70 mg m^−2^ were planned for successive patient cohorts (dose levels 2 and 3, respectively). In the case that dose level 1 was not acceptable, docetaxel 40 mg m^−2^ (dose level 0) would be explored.

At least three patients were to be started at dose level 1. If all three patients experienced DLTs, this dose level was determined to be the MTD, and dose level 0 would be explored. If two or fewer patients had DLT, an additional three patients were to be treated at the same dose level. If three or more than six patients treated at dose level 1 had DLTs, this dose level was determined to be the MTD, and dose level 0 would be explored. Dose escalation was planned until the MTD was reached, in which case the next lower dose level would be considered for further evaluation in the phase II part of the study. If three or more patients treated at dose level 0 experienced DLT, the regimen would be deemed not feasible.

Dose-limiting toxicitys were defined as follows: (1) grade 4 neutropenia lasting for 5 days or longer; (2) grade 4 neutropenia with fever (⩾38.5°C); (3) grade 4 thrombocytopenia; (4) grade 3/4 nonhaematological toxicities other than nausea/vomiting, anorexia, and general fatigue; and (5) any grade 4 haematological toxicity during S-1 administration. Assessment of DLTs was conducted only in the first treatment cycle. An independent data monitoring committee evaluated the safety results for each dose level and determined the MTD and RD.

### Phase II part

#### Study treatment

Patients received the combination treatment with the RD of docetaxel on day 1 and oral S-1 40 mg m^−2^ twice daily in accordance with the treatment regimen described above. Cycles were repeated every 4 weeks for up to six cycles unless disease progression or unacceptable toxicity was observed. The protocol did not specify rules for interruption and resumption of S1 within each cycle. Such decisions were based on the clinical judgment of the investigator.

Chemotherapy was withheld for the following toxicities: (1) neutrophil count <1500 mm^−3^ or platelet count <100 000 mm^−3^; (2) aspartate aminotransferase or alanine aminotransferase >2.5 times ULN, or total serum bilirubin >1.5 mg dl^−1^; (3) body temperature ⩾38°C; (4) performance status ⩾2; (5) diarrhoea grade 2 or more; (6) neuropathy grade 2 or more; (7) oedema grade 2 or more. Treatment was restarted after recovery to baseline. If patients did not recover from these toxicities within 28 days of the last administration of S-1, they were withdrawn from the study.

If any of the following toxicities was observed, the dose of docetaxel was reduced by one level (phase I part) or by 10 mg m^−2^ (phase II part): (1) grade 3/4 neutropenia with fever (>38.5°C); (2) haemorrhage with grade 3/4 thrombocytopenia or requirement for a platelet transfusion; and (3) grade 3/4 nonhaematological toxicity other than nausea/vomiting, anorexia, general fatigue, and hypersensitivity. The dose of S-1 could be reduced by 20 mg per day if any of these toxicities were observed after the dose reduction of docetaxel or the RD was determined to be level 0.

#### Supportive care

Throughout this study, the prophylactic administration of granulocyte colony-stimulating factor (G-CSF), antiemetic agents, corticosteroids including premedication to docetaxel, or antihistamines was not allowed during the first treatment cycle. Use of these agents was allowed as secondary prevention of symptoms in all subsequent cycles.

#### Outcome measures

Tumour response was assessed according to the Response Evaluation Criteria in Solid Tumors (Therasse *et al*, 2000). Assessments by imaging studies were repeated every 4 weeks during the study. Progression-free survival was defined as the time from registration until objective tumour progression or death (censored at second-line chemotherapy) and overall survival was defined as the time from registration until death from any cause (censored at the time of last visit in patients who were lost to –follow up). Progression-free and overall survival, and 1-year survival rates were estimated using the Kaplan–Meier method. Adverse events were graded according to the National Cancer Institute Common Toxicity Criteria (version 2). Haematological and biochemical tests, performance status and clinical assessment of symptoms were monitored at least every week in the phase I part and every 2 weeks in the phase II part. The relative dose intensity (DI) was calculated as follows: actual DI=total dose (mg m^−2^)/treatment period (weeks); planned DI (PDI)=originally planned cumulative dose in the first course (mg m^−2^)/4 weeks; and relative DI=DI/PDI.

#### Statistical methods

The design of this study was based on a binominal distribution with no planned interim analysis. For the phase II part of this study, the primary end point was to determine the ORR. Assuming a null hypothesis of a 35% ORR and an alternative hypothesis of a 55% ORR, with one-sided type I error=0.05 and type II error=0.2, it was necessary to enrol a minimum 45 patients at the RD, including those treated at the RD during the phase I part of the study. All analyses were performed using SAS® version 8.2 (SAS Institute Inc., Cary, NC, USA).

## RESULTS

Between September 2002 and June 2004, a total of 50 patients were enrolled into the study. Nine patients (three in level 1 and six in level 0) were enrolled into the phase I part and 41 patients into the phase II part. Of the 41 patients enrolled into the phase II part of the study, one patient did not receive either docetaxel or S-1 because her disease had progressed rapidly and she could not take drugs orally before the initiation of treatment. This patient was excluded from all analyses according to the Full Analysis Set principle. Therefore, the population evaluable for efficacy and safety in the phase II analysis included six patients treated at the RD during the phase I part and 40 patients treated during the phase II part of the study.

### Phase I part

The first cohort of three patients received docetaxel 50 mg m^−2^ combined with S-1 40 mg m^−2^ twice daily (dose level 1). Among these patients, one experienced grade 3 infection associated with grade 3 neutropenia on day 11, and two had grade 4 neutropenia on day 8 during S-1 administration. As all three patients treated at dose level 1 were deemed to have a DLT, the next cohort of patients was treated at dose level 0 (docetaxel 40 mg m^−2^). A total of six patients received 1–6 cycles of treatment at level 0. Among these, only one developed a DLT (grade 3 infection without neutropenia). From these results, the MTD and RD were determined to be level 1 and level 0, respectively.

### Phase II part

#### Patients

Baseline characteristics of the 46 patients treated at the RD are shown in Table 1. Median age was 65 years (range, 42–79). All patients had at least one measurable lesion. Thirty-four patients (74%) had primary gastric lesions. Twenty-two patients (48%) had hepatic metastases. Sixteen patients (35%) had received a prior chemotherapy including 5-FU-containing regimens, irinotecan plus cisplatin or oral fluoropyrimidines.

#### Study treatment

A total of 165 cycles were administered, with a median of three cycles (range, 1–6). The median cumulative doses of docetaxel and S-1 were 120 mg m^−2^ (range, 40–240) and 3098 mg m^−2^ (range, 204–6368), respectively. Median relative dose intensities of docetaxel and S-1 were 99% (range, 75–101) and 82% (range, 18–97), respectively. Thirty-six patients (78%) who failed study treatment received the next-line chemotherapy. The main regimen was cisplatin plus irinotecan (17/36, 47%).

#### Efficacy

**Figure 1:**
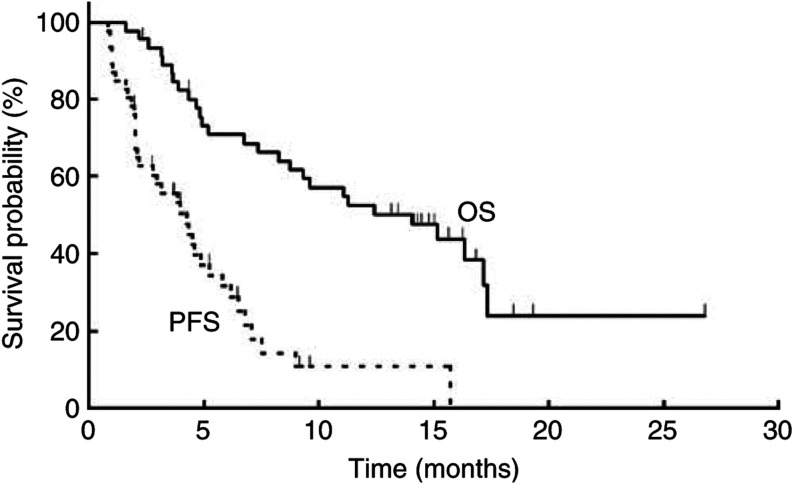
The cumulative probability of survivals. The solid and dotted lines present overall and progression-free survivals estimated by the Kaplan–Meier method in 46 patients, respectively.

Tumour response results are shown in Table 2. The ORR was 46% (95% confidence interval ((CI), 31–61) with two patients (4%) showing a complete response and 19 (41%) a partial response. Figure 1 shows overall and progression-free survival. At a median follow-up of 12 months (range, 2–27), the median progression-free and estimated overall survival times were 4.2 months (95% CI, 2.2–5.2) and 14 months (95% CI, 8.3–17.3), respectively. The 1-year survival rate was 53% (95% CI, 38–67).

#### Safety

Table 3 summarises adverse events observed during the phase II part of this study. The most common grade 3/4 haematological toxicities were neutropenia (31 patients, 67%) and leucopenia (19 patients, 41%). Among a total of 165 cycles, the median time from the treatment start to nadir was 14 days (range, 3–29) and the median time from nadir to recovery was 15 days (range, 2–27). The study treatments were delayed for the following reasons: neutropenia (6/165 cycles, 4%), thrombocytopenia (3/165, 2%), worsening of PS (2/165, 1%), and fever (1/165, 1%). The doses of S-1 were reduced in 16 patients (35%) mainly due to grade 4 neutropenia and grade 3 anorexia. Treatment with G-CSF as secondary prophylaxis for neutropenia was administered in 23 cycles (14%). The most common grade 3/4 nonhaematological toxicity was anorexia (10 patients, 22%). Fever and infection were observed in 10 patients (22%, grade 1 or 2) and two patients (4%, all grade 3), respectively. Oedema (grade 1) was observed in only three patients (7%), despite the absence of prophylactic corticosteroids or antihistamines. The majority of the nonhaematological toxicities were relatively mild. No treatment-related deaths were observed.

In five patients, combination treatment was discontinued due to the following adverse events: grade 4 neutropenia associated with grade 3 diarrhoea, grade 4 neutropenia associated with grade 2 thrombocytopenia, grade 3 neutropenia associated with unrecovered grade 2 anaemia, grade 4 cerebral infarction, and suspected grade 2 interstitial pneumonia.

## DISCUSSION

We evaluated the efficacy and safety of a chemotherapy combination regimen of docetaxel and S-1, two agents that separately have shown promise in the management of advanced GC. Response rates and safety data for S-1 have shown ORRs up to 49% with no grade 4 haematological toxicity (Sakata *et al*, 1998; Koizumi *et al*, 2000), while adding docetaxel to a standard chemotherapy regimen of cisplatin and 5-FU improves survival (TAX 325, Moiseyenko *et al*, 2005). Therefore, our hypothesis was that docetaxel combined with S-1 would confer a clinically meaningful improvement in ORR and median survival, with a manageable safety profile.

In the phase I part of our study, we identified docetaxel 40 mg m^−2^ on day 1 plus S-1 40 mg m^−2^ twice a day on days 1–14, every 28 days as the treatment schedule to recommend for further clinical evaluation. This dose of docetaxel is lower than that commonly used in the West to treat GC. However, we observed severe myelosuppression at dose level 1 (docetaxel 50 mg m^−2^), which was the MTD. In this study, the MTD was declared when all of the first three patients in a cohort experienced DLT or when at least three of six patients enrolled in a cohort experienced DLT. Although this definition differs from that employed in other studies, it is commonly accepted in Japan. Our use of this definition allowed us to find a RD that was tolerable for most patients enrolled in the phase II portion of this study.

We speculate the reason for the lower dose of docetaxel may be that the pharmacokinetic parameters (AUC and *C*_max_) of 5-FU increase according to the dose of docetaxel. For example, the respective mean AUC and *C*_max_ of 5-FU were 522.5 ng h ml^−1^ and 100.3 ng ml^−1^ with docetaxel 40 mg m^−2^ and 857.2 ng h ml^−1^ and 155.8 ng ml^−1^, with docetaxel 50 mg m^−2^ (Yoshida *et al*, 2004). Synergy of this combination has been reported *in vitro*, suggesting that biochemical modulation of the two drugs occurs. This synergy may also result in increased toxicity (Wada *et al*, 2006). Perhaps higher doses of docetaxel would have conferred a greater response rate, but such doses were not feasible in our study population. Preliminary reports have described alternative dosing schedules for docetaxel in combination with S1 for the treatment of advanced GC. These have explored docetaxel dosing intervals ranging from every week to every 4 weeks with relative dose intensities from 10 to 20 mg m^−2^ per week. However, because final reports of these studies are not yet available, the feasibility and efficacy of these alternative regimens remains unknown (Yoshida *et al*, 2004; Kim *et al*, 2006; Park *et al*, 2006; Rino *et al*, 2006; Satoh *et al*, 2006; Takahashi *et al*, 2006).

In the phase II part of this study, we demonstrated that for patients with advanced GC, this regimen conferred an ORR of 46% and an estimated median survival of 14 months, with acceptable toxicity. The 46% ORR observed in this study was slightly lower than expected. We anticipated an ORR of 55% because results from phase II studies in Japan showed ORRs of 22% with docetaxel monotherapy (Taguchi *et al*, 1998; Mai *et al*, 1999) and 45% with S-1 (Sakata *et al*, 1998; Koizumi *et al*, 2000). A possible explanation is that we included a significant number of patients with liver metastasis (22/46, 47.8%) and intestinal-type histology (29/46, 63%), and many had previous exposure to chemotherapy (16/46, 34.8%). All of these features are associated with decreased response rates to chemotherapy (Taguchi *et al*, 1998, Mai *et al*, 1999, Koizumi *et al*, 2000, Ajani *et al*, 2005). Yet despite these unfavourable baseline characteristics, the combination of docetaxel and S-1 conferred an ORR that was similar to those obtained with other combination regimens (Ajani *et al*, 2005; Thuss-Patience *et al*, 2005).

At a median follow-up of 12 months, the median overall survival was estimated to be 14 months. Although cross-study comparisons should be taken with caution, the survival effect observed in our study compares favourably with other chemotherapy regimens, such as docetaxel (6–8 months (Taguchi *et al*, 1998; Mai *et al*, 1999)) or S-1 (7–8 months (Sakata *et al*, 1998; Koizumi *et al*, 2000)), or the combinations DC (11 months), DF (10 months), DCF (10 months), ECF (10 months) or FOLFOX-4 (11 months) (Ajani *et al*, 2005; De Vita *et al*, 2005; Thuss-Patience *et al*, 2005).

Patients enrolled in our study were, in some respects, less likely to respond to chemotherapy than patients in these other trials. All patients in our study had metastatic disease, 65% were chemo-naive, and 48% had liver metastases. In studies of DCF, ECF, and FOLFOX-4, the proportions of chemo-naive patients were 100, 100, and 84%, respectively; the rates of metastatic disease were 95, 98, and 92%, respectively; and the incidences of liver metastases were 80% (including peritoneal), 36, and 62%, respectively. Despite this, the ORR and survival observed in our study appeared higher than those reported for other combination regimens studied in more favourable populations. At a median follow-up of 12 months, the estimated median survival is 14 months, but this may improve over time.

Although survival data appear promising, there are limitations. Progression-free survival in this study was similar to those reported with conventional reference regimens containing 5-FU and cisplatin (Vanhoefer *et al*, 2000; Ohtsu *et al*, 2003). In addition, the median survival observed in our study may be partly influenced by poststudy chemotherapy, which was administered to 78% of patients.

The docetaxel/S1 regimen reported here was well tolerated and toxicities were manageable. The most common haematological toxicities were neutropenia and leucopenia. The incidence of grade 3/4 neutropenia was similar to those previously reported with docetaxel monotherapy (Sulkes *et al*, 1994; Einzig *et al*, 1996; Taguchi *et al*, 1998; Mai *et al*, 1999). Despite the lack of corticosteroid prophylaxis, only three patients developed fluid retention (all grade 1), and no patients had a hypersensitivity reaction. The majority of nonhaematological toxicities were mild.

The docetaxel/S1 regimen used in this study yielded a promising median survival time and manageable safety profile. Based partly on these results, a phase III study comparing S-1 alone to docetaxel plus S-1 in patients with advanced GC has begun enrolment in Japan to evaluate whether adding docetaxel to S1 improves clinical benefit.

In conclusion, the combination of docetaxel and S-1 is an active and well-tolerated regimen in patients with advanced GC. It is worthwhile to assess the survival benefit and quality of life in a phase III trial of this combination in advanced GC to establish the new regimen for the outpatient setting.

## Figures and Tables

**Table 1:** Baseline characteristics of 46 patients* in the phase II part

**Characteristic**	**Number of patients (%)**
*Sex*	
Male	31 (67)
Female	15 (33)
	
*ECOG performance status*	
0	29 (63)
1	17 (37)
	
*Histology*	
Intestinal	29 (63)
Diffuse	17 (37)
	
*Metastatic sites*	
Lymph nodes	35 (76)
Liver	22 (48)
Ovary	1 (2)
Spleen	1 (2)
Rectum	1 (2)
Adrenal gland	1 (2)
Gastric remnant	1 (2)
	
*No of metastatic sites*	
1	31 (67)
2	14 (30)
3	1 (2)
	
*Prior therapy*	
None	25 (54)
Any prior therapy	21 (46)
Surgery	13 (28)
Chemotherapy	16 (35)

ECOG=Eastern Cooperative Oncology Group.

* Six patients treated at the recommended docetaxel dose of 40 mg m^−2^ in the phase I part were included in analysis.

**Table 2:** Tumour responses in the phase II part

	***N***	**Response rate (%)**
Total	46	46^a^
Sub-group analyses by		
		
*Age*		
<65	23	43
65⩽	23	48
		
*Histology*		
Intestinal	29	41
Diffuse	17	53
		
*Prior chemotherapy*		
Not received	30	50
Received	16	38
		
*Liver metastasis*		
Absent	24	67
Present	22	23

a 95% confidence interval=31–61%.

**Table 3:** Adverse events observed in 46 patients

	**Number of patients grade^a^**		
**Adverse events**	**3**	**4**	**Number of patients(%) grade 3 or 4**
Neutropenia	15	16	31 (67)
Leucopenia	16	3	19 (41)
Anaemia	9	1	10 (22)
Anorexia	10	0	10 (22)
Hyponatremia	8	0	8 (17)
Nausea	4	0	4 (9)
Vomiting	1	0	1 (2)
Stomatitis	3	0	3 (7)
Diarrhoea	2	0	2 (4)
AST elevation	3	0	3 (7)
Infection	2	0	2 (4)

AST=aspartate aminotransferase.

a National Cancer Institute common toxicity criteria (version 2).
